# Is a Register for Nurses Desirable?

**Published:** 1889-01-05

**Authors:** 


### The Hospital

A Weekly Institutional Journal of

**Science, Medicine, Nursing, and Philanthropy.**

*For Hospitals, Asylums, Medical Practitioners, Students, Nurses, Families, Parishes, Congregations, and the Charitable Public.*

Vol. V. No. 119.] [January 5, 1889.

### Is a Register for Nurses Desirable?

At the beginning of a new year both the public who employ trained nurses and nurses themselves may very properly follow the example of houses of business and "take stock." We do not propose, however, in the present article to offer a discursive review of the progress of the nursing profession during the past twelve months, but rather to concentrate attention on one single question, but that a question of prime importance. Nurses are not only justified in making all possible efforts to increase the efficiency and raise the status of their profession, but it is their imperative duty to do so. The better the nurse the more will she help the sick and the worthier will be her influence on the well. Every profession and calling should be animated by a noble *esprit de corps*, and each individual member of it should strive to promote the excellence and prosperity of the whole. It is with these convictions clearly defined in the mind, and a long and intimate acquaintance with the work, the difficulties, the merits, and the hopes of trained nurses, that we approach the subject which is indicated by the title of this article: "Is a Register for Nurses Desirable?" Most assuredly it is. Every hospital-trained nurse should possess a dipolma or certificate of registration from the hospital in which she has been trained. Such certificate ought to be, and practically is in all the leading hospitals in the country, an official document, signed by the executive authorities of the institution to which she belongs, embodying the period the nurse has worked and the manner in which she has performed her duties. The handy woman, the midwife, the specialist, and the monthly nurse possess no such passport to public confidence unless they also have served their appointed time in a public hospital. But when we come to inquire the length of the term the nurse has served and the character of her curriculum, we unfortunately encounter grave differences even in the practice of the best known hospitals; and until these differences are adjusted so as to place all in accord, there is little hope of any collective system of nurse-registration being sanctioned by the Legislature. If such an organisation can be seriously contemplated, it is clear that the initiative must be taken by the training schools themselves; for it is hardly to be surmised that the hospitals would be dictated to on such an important matter as the educational training of their nurses by an unaccredited body outside their jurisdiction.

The Hospitals Association, with whom this question of nurse-registration originated, after much consideration, resolved that it was imperative to follow the lead of the persons most directly concerned; that is, the medical and executive authorities of the leading hospitals in London and the provinces, where systems of nurse-training were known to be in force. With this view, they addressed a series of questions bearing on the subject to the representatives of over thirty hospitals, and the conflict of opinion expressed in the answers left little doubt in the mind of practical persons that the time had not arrived for legislative action. It is true that only a bare majority of the institutions referred to were averse to collective registration, but among these, with one exception, were the leading hospitals of the metropolis, together with the chief hospitals of Liverpool, Manchester, Glasgow, and Edinburgh, all of which had been early pioneers in the movement of nurse reform. The reasons for their opposition to what many considered a popular measure were of such importance and weight that we may be excused for again referring to them. It was shown that each training school of acknowledged repute already possessed a register of its own, and furnished each of its nurses with a registration document in the form of an authorised certificate after she had fulfilled her engagement. A large proportion, and these the best-known hospitals, considered they would lose rather than gain by amalgamation with others of inferior prestige, and which are increasing in number from year to year in every direction. A central organisation it was thought could not fail to affect the individuality and the healthy rivalry which now exists by reducing the best hospitals to a level with others wanting in the same facilities for training. It was also strongly impressed on the committee who had charge of the inquiry that, considering the various objects, commercial and charitable, for which nurses are required, and the far from general unanimity existing in their own ranks as to the nature and expediency of a system of collective registration, it would be injudicious, to say the least, for any self-constituted body to undertake of itself such an important obligation. The advantages of each hospital and training school preserving its autonomy are only too apparent, and it is to be deplored that, notwithstanding the results of the inquiry, persistent efforts continue to be made by a society with an imposing name to delude nurses into the belief that if they subscribe to it annually they may become registered, and thereby participate in some shadowy benefits in the future.

We are led to make these observations by a sincere desire to help nurses, and in no unfriendly spirit.

Every effort on their part to form a friendly or mutual benefit society, or a professional union, whereby the stronger may help the weaker, will always have our warm support. But we caution them not to be led astray by specious promises which those better informed than themselves know cannot be fulfilled. The National Pension Fund for Nurses, backed as it is by gentlemen who by their princely munificence have shown what interest they take in the whole body of nurses, has, by its articles of association, already secured the legal right to provide and maintain a register for trained and certificated nurses; and the executive will not be slow to put it into execution so soon as the general opinion of the nursing profession and of the training schools shall point the way. We are not without hopes, which we trust may not be frustrated by injudicious and unpractical enthusiasts, that such a desideratum may yet be obtained. The medical and lay authorities of all hospitals are bent on making this important part of the service as efficient as their funds will enable them, while the smaller institutions, and even poor-law infirmaries, are engaged in initiating reforms in their nursing with the view of approaching the level of the great hospitals. In addition to the three years' probation, prior to a nurse being eligible for her certificate, it is now generally considered necessary that she should attend a few elementary lectures on subjects cognate with her vocation, to induce her to take a more intelligent interest in it. With this it may be expected that, before receiving her certificate, she will be called upon to undergo some system of examination to test her knowledge and manipulative powers, but as these matters come daily under the inspection of her employers, we are not disposed to place too much reliance on the ordeal.

It is argued, and with much reason, that a clever woman, after a few weeks of hospital life and an attentive perusal of some nursing literature, may become sufficiently conversant with its technicalities to be able to write a book and even to lecture on the subject, but after all may turn out to be a very incompetent nurse. Nevertheless it would greatly help to raise the status of the professional nurse if the leading hospitals concurred in an arrangement by which she could give some intellectual evidence of her training apart from the fact that she had spent three or more years in their employ. Something of the kind has been attempted at more than one of the training schools, but there is great lack of uniformity, and instances must be rare indeed where a nurse has been deprived of her registered certificate by failing to answer technical questions when her character is otherwise unexceptionable. A certificate of registration should under no circumstances be had for money. Such a document, to which every nurse is entitled by virtue of her services and good conduct, ought to be free from every suspicion of commercial advantage to the Registration Office. An annual registration fee would be an unjustifiable extortion, and nurses must resist it with all their might if it should be proposed. As a rule nurses are underpaid for their work; the institutions employing them reap the benefit of their earnings, especially in the early stages, and it would be a cruel injustice to withhold from them for money considerations what must be considered as their right. The question, as we have said, is one of profound importance to the nursing profession, and we shall return to it from time to time, with the view of helping to form an intelligent public opinion, and of furthering the development of a comprehensive and practical scheme which shall commend itself to all those who, by knowledge, experience and personal interest are capable of forming sound judgments on the subject.

